# PSMA PET imaging in the diagnosis and management of prostate cancer

**DOI:** 10.1007/s00261-023-04002-z

**Published:** 2023-07-26

**Authors:** Sina Houshmand, Courtney Lawhn-Heath, Spencer Behr

**Affiliations:** Department of Radiology and Biomedical Imaging, University of California San Francisco, San Francisco, California USA

**Keywords:** PSMA, Prostate cancer, PET, Biochemical recurrence

## Abstract

Prostate cancer is the second leading cause of cancer-related deaths in men in the United States. Imaging techniques such as CT, MRI, and bone scans have traditionally been used for diagnosis and staging. Molecular imaging modalities targeting the prostate-specific membrane antigen (PSMA) have recently gained attention due to their high affinity and accuracy. PSMA PET has been combined with other modalities such as multiparametric MRI for better diagnostic and prognostic performance. PSMA imaging has been studied at different clinical settings with a wide range of disease aggressiveness. In this review we will explore the role of PSMA PET in high-risk prostate cancer staging, biochemical recurrence, and castration-resistant prostate cancer. The primary focus of this review article is to examine the latest developments in the use of PSMA imaging and emphasize the clinical situations where its effectiveness has been demonstrated to significantly impact the treatment of prostate cancer. In addition, we will touch upon the potential future advancements of PSMA PET imaging and its evolving significance in the management of prostate cancer.

**Graphical abstract:**

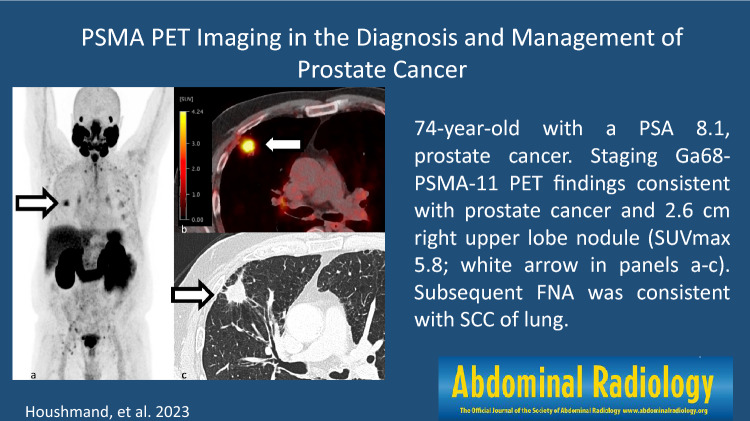

## Introduction

Prostate cancer is the most common cancer in men in the United States and second leading cause of cancer-related death in men [1]. CT, MRI and bone scan are the conventionally used imaging techniques for diagnosis, staging and restaging of prostate cancer [2]. Prostate specific membrane antigen (PSMA) is a membrane glycoprotein, expressed 100 to 1000 times higher on prostate cancer cells compared to normal prostate [3]. Imaging agents targeting PSMA are now widely available and have drawn attention due to their high affinity and accuracy in diagnosis, staging, restaging of prostate cancer [4]. Numerous PSMA agents using different radiotracers and imaging modalities such as SPECT and PET have been developed and studied in the literature [5, 6]. PSMA PET has been combined with other modalities such as multiparametric MRI, for a better diagnostic and prognostic performance [5].

The role of PSMA has been studied at different clinical settings with a wide range of disease aggressiveness [4]. Recently, a multidisciplinary panel of healthcare providers and prostate cancer imaging experts developed the appropriate use criteria for PSMA imaging [2]. Patients with newly diagnosed unfavorable intermediate, high, or very-high-risk prostate cancer, biochemical recurrence, and castration-resistant prostate cancer (CRPC) represent the scenarios with the highest scores for PSMA imaging utilization (see Table 1) [2].

**Table 1:** Updated appropriate use criteria for use of PSMA in prostate cancer patients with 12 different clinical scenarios [60]

Scenario no.	Description	Appropriateness	Score
1	Patients with suspected prostate cancer (e.g., high/rising PSA levels, abnormal digital rectal examination results) evaluated for targeted biopsy and detection of intraprostatic tumor	Rarely appropriate	3
2	Patients with very low, low, and favorable intermediate-risk prostate cancer	Rarely appropriate	2
3	Newly diagnosed unfavorable intermediate, high-risk, or very-high-risk prostate cancer	Appropriate	8
4	Newly diagnosed unfavorable intermediate, high-risk, or very-high-risk prostate cancer with negative/equivocal or oligometastatic disease on conventional imaging	Appropriate	8
5	Newly diagnosed prostate cancer with widespread metastatic disease on conventional imaging	May be appropriate	4
6	PSA persistence or PSA rise from undetectable level after radical prostatectomy	Appropriate	9
7	PSA rise above nadir after definitive radiotherapy	Appropriate	9
8	PSA rise after focal therapy of the primary tumor	May be appropriate	5
9	nmCRPC (M0) on conventional imaging	Appropriate	7
10	Posttreatment PSA rise in the mCRPC setting in a patient not being considered for PSMA-targeted radioligand therapy	May be appropriate	5
11	Evaluation of eligibility for patients being considered for PSMA-targeted radioligand therapy	Appropriate	9
12	Evaluation of response to therapy	May be appropriate	5

This review article focuses on recent advances in the applications of PSMA imaging and highlights the clinical scenarios where its effectiveness has been proven to significantly alter the management of prostate cancer.

## PSMA-targeting radioligands

Since its discovery in 1987, multiple iterations of PSMA-targeting molecules for the purpose of diagnosis and treatment of prostate cancer have been developed [7]. For a long time, prostascint (ProstaScint, capromab pendetide; EUSA Pharma) which is a radiolabeled anti PSMA antibody, was the only agent which was FDA approved [8]. Emergence of small molecule PSMA ligands has enabled a more efficient targeting by attaching to an extracellular domain of the PSMA molecule [8]. While PSMA expression tends to increase with aggressiveness, it has been shown to decrease in highly de-differentiated tumors [9]. In addition, higher PSMA uptake is associated with castration resistance and disease progression [3]. Among numerous PSMA-targeting molecules, 68Ga‐PSMA‐11 and 18F-DCFPyL have recently received FDA approval [2]. These new generation PSMA-targeting ligands are made in form of small molecules which clear more rapidly from blood and are more efficient with better tumor to background ratio compared to the older antibody-based counterparts [10]. There is a long list of PSMA-targeting agents which are currently in active clinical trials [11] such as 18F-PSMA-1007, 68Ga-PSMA-617, 68Ga-PSMA-I&T, and 18F-rhPSMA-7.3 [5]. 18F-rhPSMA-7.3 is a radiohybrid radiotracer with high affinity to PSMA and reported lower urinary excretion compared to 68Ga-PSMA-11 and 18F-DCFPyL, with favorable results in the SPOTLIGHT trial [12].

## PSMA scan

**Fig. 1:**
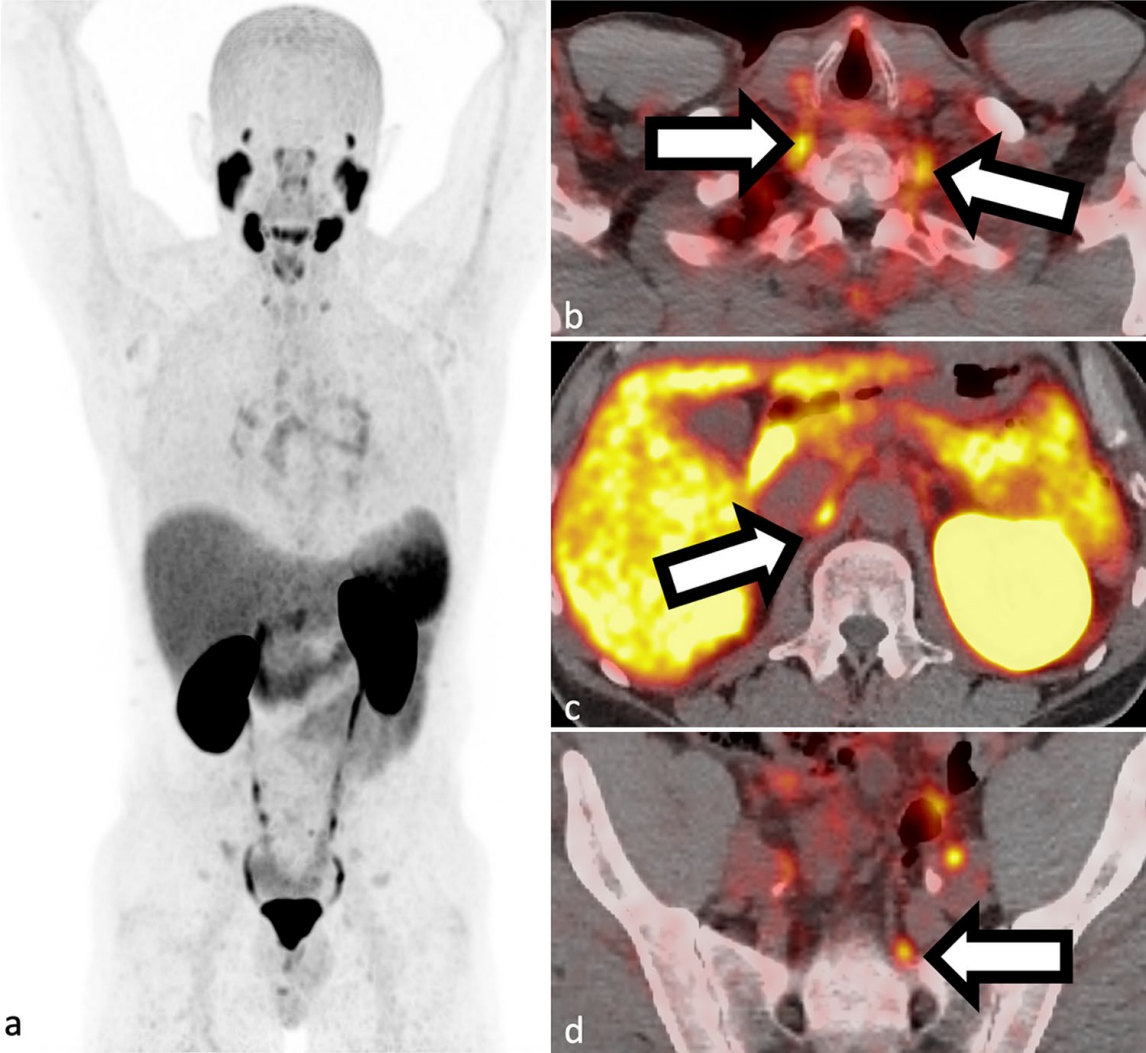
**a–d** Example of normal 68Ga-PSMA-11 PET biodistribution in whole-body maximum intensity projection (**a** SUV scaled at 0–10) with cervical (**b**), celiac (**c**) and presacral (**d**) ganglion uptake

The European Association of Nuclear Medicine (EANM) and the Society of Nuclear Medicine and Molecular Imaging’s (SNMMI) joint procedure standard for 68Ga-PSMA-11 [13] recommends a 60-minute uptake time with voiding immediately before imaging to minimize bladder uptake. Delayed imaging is recommended in BCR patients with low PSA (< 1ng/mL) [14]. To avoid artifact from residual activity in collecting system, administration of furosemide or hyper hydration has been suggested [15]. Intravenous contrast administration and imaging at urographic phase has also been a solution for delineation of urinary collecting system from tumor burden [16]. Conventionally accepted uptake time for 18F-PSMA compounds range between 60 and 120 minutes [17]. The normal biodistribution of PSMA is seen on Fig. 1. Frequently, either cervical, celiac and/or presacral ganglia can be seen. It is important to know that these are variant of normal biodistribution. Additionally, PSMA shows specific uptake in astrocytes, proximal renal tubules, small intestine, salivary and lacrimal gland [18].

The estimated coefficient for effective dose from PSMA PET ligands ranges between 0.0116 and 0.022 mSv/MBq, resulting in calculated effective doses ranging between 3.4 and 6.6 mSv depending on radioligand type and injection dose [13]. These numbers are comparable to other PET radiotracers such as 18F-choline (0.01mSv/MBq) [19]. Kidneys and lacrimal glands receive the highest absorbed dose in 68Ga-PSMA PET [19]. Radiation exposure from the CT portion of the scan varies and depends on the protocol with effective dose ranging from 1 to 20 mSv [13].

## PSMA vs. other compounds

### Choline PET

The increased turnover and production of phosphatidyl choline in cellular membranes is the basis of this radiotracer [20]. Choline is either conjugated with 11C or 18F. Studies have shown promising results in patients with BCR despite lower detection rates compared to PSMA PET, with the difference more pronounced on lower PSA levels [21, 22]. Although FDA approved in 2012 for BCR, short half-life of 11C limits availability of this radiotracer to centers with on-site cyclotron. 18F Choline has a longer half-life, enabling wider adoption. This radiotracer was not adopted in the United States due to regulatory issues [4].

### Fluciclovine PET

Fluciclovine PET targets amino acid transporters for L-lerucine[4]. Fluciclovine was shown to be at least comparable to PSMA in the earlier studies of patients with advanced disease [23, 24]. It has been FDA approved in 2016 for BCR of prostate cancer. In the more recent clinical trials, Fluciclovine was compared to PSMA in a head-to-head prospective trial of 50 patients with BCR and low PSA (< 2.0 ng/mL) [25]. The detection rate for PSMA was at least twice (56% vs. 26%).

## Staging of high-risk prostate cancer

**Fig. 2:**
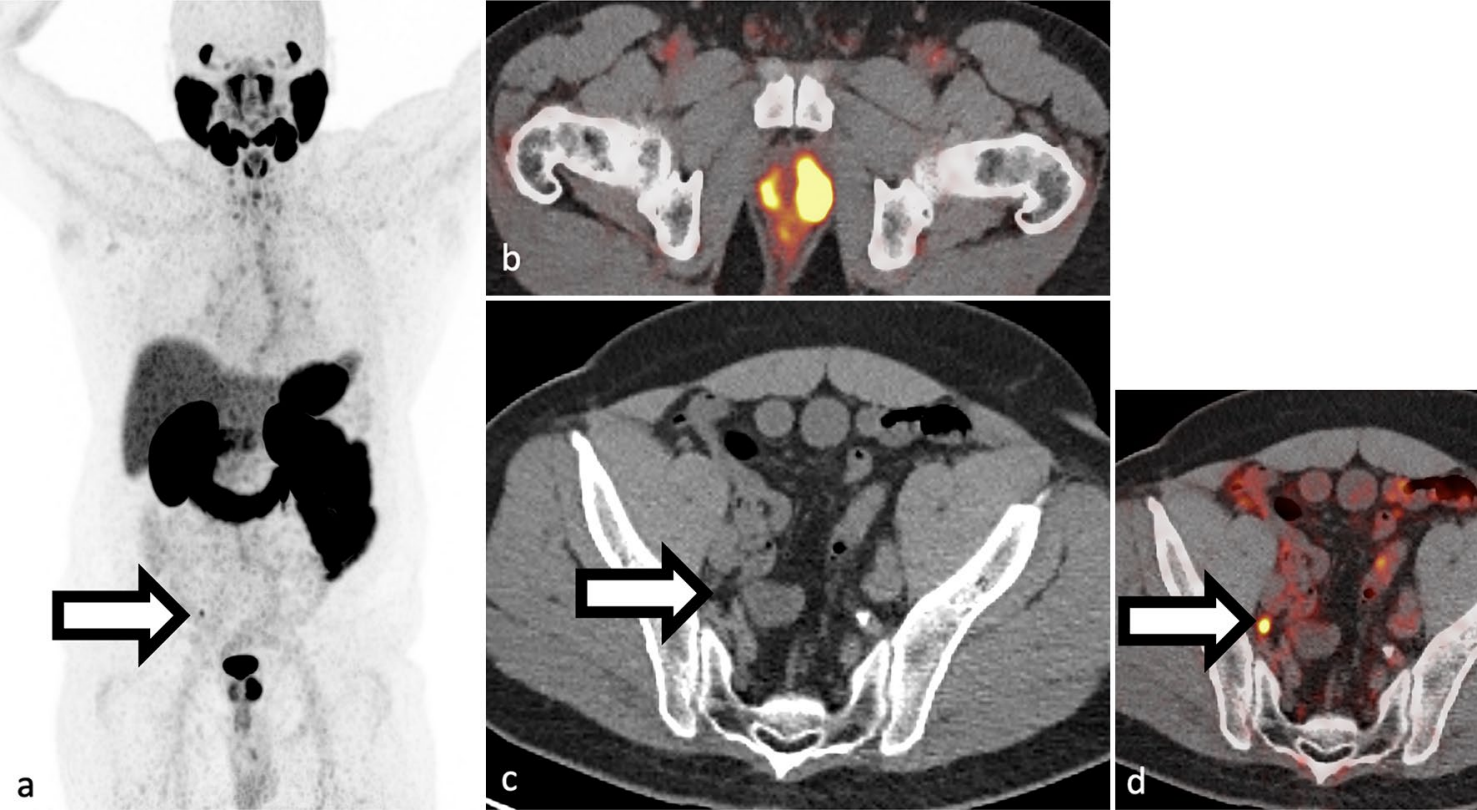
74-year-old with high-risk prostate cancer. Pre-surgical planning 68Ga-PSMA-11 PET/CT performed. Single PSMA avid 2 mm right internal iliac chain lymph node (white arrow in panels a and d; SUVmax 4.5), not well seen on CT (white arrow on panel c). Maximum intensity projection PET **a** is scaled at 0–5 SUV

The initial staging of prostate cancer is a crucial step in determining the most appropriate treatment plan, including options such as prostatectomy, radiation therapy with or without local lymph node treatment, or systemic therapy [4] (Fig 2). Risk stratification schemes, based on criteria such as histologic pattern, PSA level, and clinical stage, have been developed to optimize treatment planning [26]. The latest update on cancer center network guideline defines a 5-tier risk stratification method for clinically localized prostate cancer [27]. Imaging is generally not indicated for low and very low-risk prostate cancer, as it is unlikely to extend beyond the prostate [2, 27].

The majority of studies examining the role of PSMA in prostate cancer staging have reported favorable specificity and predictive values. Hope et al. [28] in their prospective multicenter single-arm phase 3 trial of 277 patients with high-risk prostate cancer with Ga68-PSMA-11 PET prior to prostatectomy, 27% of patients were positive for pelvic lymph node metastasis with sensitivity of 40%, specificity of 95%, positive predictive value of 75%, and negative predictive value of 81%. The findings of this study formed the foundation for new drug application of 68Ga-PSMA-11. The OSPREY study [29] was a multicenter prospective clinical trial with 18F-DCFPyL that looked at 252 patients undergoing radical prostatectomy, which found a median specificity of 97.9% and a not much favorable sensitivity of 40.3% was achieved for detection of pelvic lymph nodes. The positive predictive value was 86.7% and negative predictive value was 83.2% [29].

Some studies have compared the performance of PSMA PET versus conventional imaging. In the proPSMA study, a multicentric randomized clinical trial of 302 patients aimed at investigating the accuracy of Ga68-PSMA-11 in staging prostate cancer in high-risk patients at initial presentation compared to conventional imaging, PSMA PET showed superior accuracy (92% compared to 65% in conventional imaging), sensitivity (85% versus 38%) [30]. Another study of 160 high-risk prostate cancer patients evaluated with F18-DCFPyL for initial staging, 90% of patients with distant metastasis were correctly identified, where 48% of these patients did not have enlarged lymph node on CT [31]. Nearly all the patients with evidence of metastasis on CT, were found to have additional sites on F18-DCFPyL [31]. Based on the results of these studies, PSMA PET appears to be superior to conventional imaging [32, 33] and appropriate for initial staging of the unfavorable intermediate, high, or very-high-risk prostate cancer [2]. A meta-analysis that evaluated 257 patients for initial evaluation of prostate cancer (primary and metastatic lymph node combined) in a per-lesion analysis, found sensitivity of 83% and specificity of 81% in diagnosis of primary prostate cancer [34].

## Biochemical recurrence

**Fig. 3:**
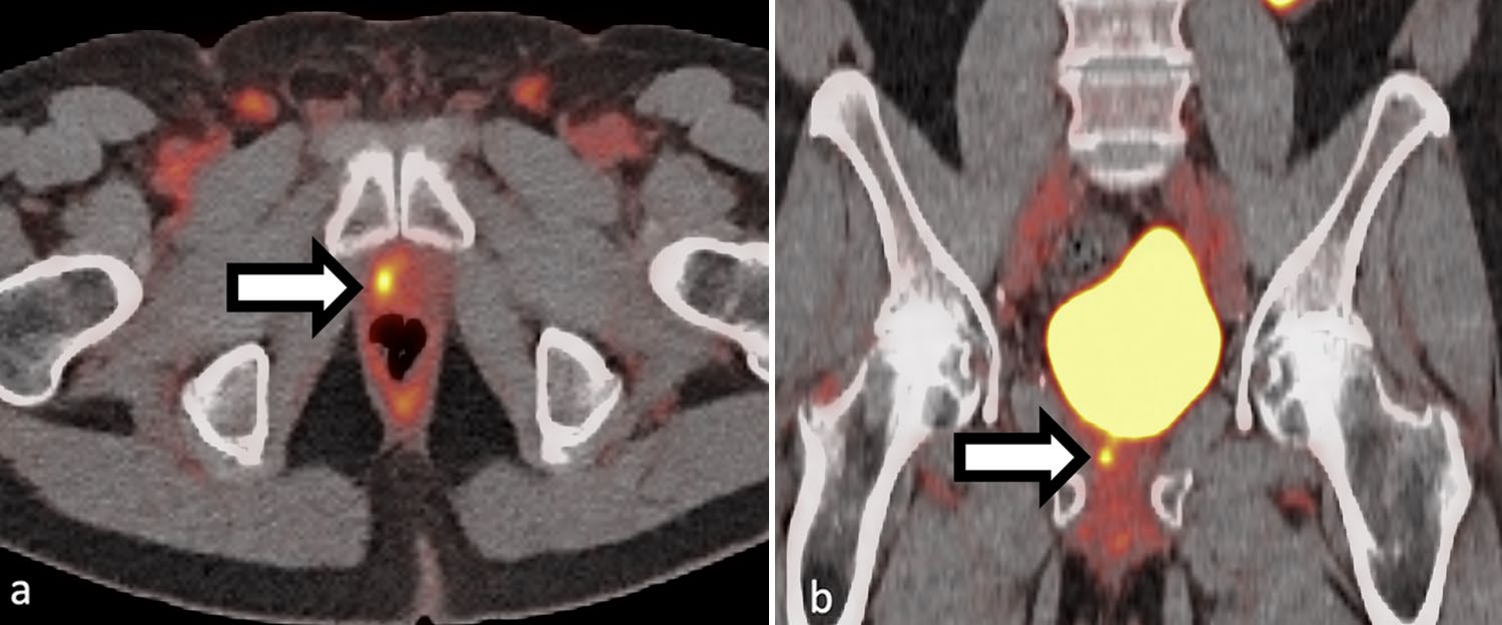
**a–b** Example of local recurrence in a 75-year-old patient presenting with slowly rising PSA after prostatectomy 20 years prior. F18-DCFPyL PET/CT shows focal uptake in the right surgical bed at the anastomosis (white arrows in panels a and b)

**Fig. 4:**
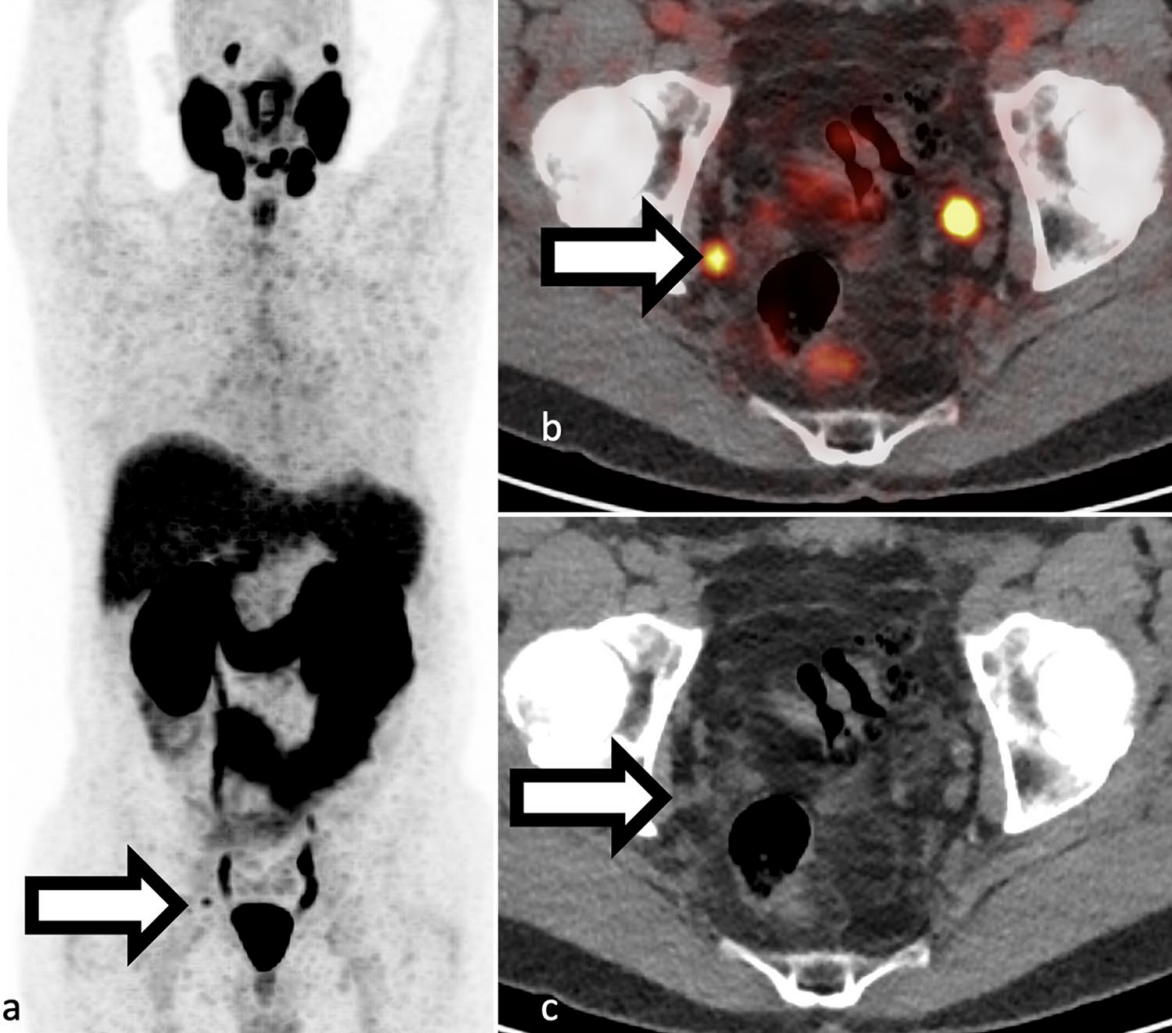
**a–c** Example of single PSMA lymph node disease in a 54-year-old with recent resection for prostate cancer with persistently elevated PSA. F18-DCFPyL PET/CT showed a single focus of PSMA uptake in right pelvis (panel a and b, SUVmax 4.4) corresponding to a 4 mm right pelvic side wall lymph node (panel c). Original preprostatectomy pathology showed up to Gleason 4+5 prostate adenocarcinoma in left prostate lobe and 10/17 cores were positive for cancer. Uptake in the left pelvis corresponds to ureteric activity. Maximum intensity projection view **a** SUV scaled at 0–5

Biochemical recurrence occurs when the serum PSA level increases to a specific threshold following definitive therapy. In radiation therapy, this threshold is more than 2.0 ng/mL above nadir, while in radical prostatectomy, it is 0.2 ng/mL on two consecutive reads [35, 36]. PSA recurrence after definitive therapy is common [37] with about 50% of patients experiencing PSA recurrence within 10 years (Figs. 3 and 4 ) [37].

Early detection of biochemical recurrence (BCR) may provide a window of opportunity for effective treatment with directed radiation therapy and/or surgery before the onset of clinical recurrence. For example, salvage radiation therapy which has been shown to be the most effective treatment plan after radical prostatectomy and BCR, is usually done without conventional imaging, potentially missing distant lesions [38]. Therefore, a sensitive imaging technique to detect disease out of expected radiation field for optimal treatment is critical for management of BCR [4]. In a recent post hoc analysis involving 270 patients, PSMA PET imaging was found to alter patient management in 19% of cases [39]. As a result, a clinical trial by Calais et al. is currently investigating the potential value of PSMA PET imaging in radiation planning for these patients [40].

There is a large body of evidence supporting use of PSMA PET in BCR [39, 41–50]. Hope et al. in their meta-analysis of 256 patients, found sensitivity of 99%, specificity of 76% in pelvic lymph nodes in patients with BCR using pathologic correlate as gold standard [51]. In a more recent systematic review and meta-analysis, Pozdnyakov et al. [50] found that PSMA PET altered treatment plans in 56.4% of the population from 34 studies (3680 patients) and resulted in BCR-free survival of 60.2% at median follow up of 20 months for 1057 patients. In another meta-analysis of 4790 patients [52], Ga68-PSMA PET was found to improve detection rates with rising PSA levels from 33% for PSA levels below 0.19 ng/mL and to 95% for PSA levels > 2.0 ng/mL. This study found high sensitivity and specificity in detection of nodal metastasis in BCR patients (75% and 99%, respectively) comparable with conventional imaging [32, 52]. PSMA PET was able to predict three-year freedom from disease progression in the multicenter clinical trial on prostate cancer patients with BCR undergoing salvage radiotherapy [44]. PSMA is now incorporated in biochemical recurrence guidelines of American Urology Association Radiographic Assessments for Detection of Advanced Recurrence (RADAR III) consensus group [53], European association of urology [54] and the NCCN guidelines for prostate cancer [55].

## Castration-resistant prostate cancer and PSMA-targeted treatment of prostate cancer

Castration resistance in prostate cancer occurs when the disease continues to progress, even in the presence of low levels of prostate-specific antigen (PSA) and despite treatment with androgen deprivation therapy. Distinguishing between metastatic and nonmetastatic castration-resistant prostate cancer (CRPC) is crucial for determining the appropriate treatment plan [2]. PSMA PET imaging is highly effective in accurately evaluating the extent of disease in both patient groups. In a multicentric retrospective study of 200 patients with CRPC, PSMA PET was able to detect metastatic disease in 55% of patients who were designated as M0 based on conventional imaging [56]. Neuroendocrine differentiation, a rare but increasingly aggressive subtype of prostate cancer, can be observed in the advanced stages of CRPC [57]. However, recent advancements in PSMA-targeted treatments for prostate cancer have demonstrated promise in effectively treating these types of prostate cancers.

**Fig. 5:**
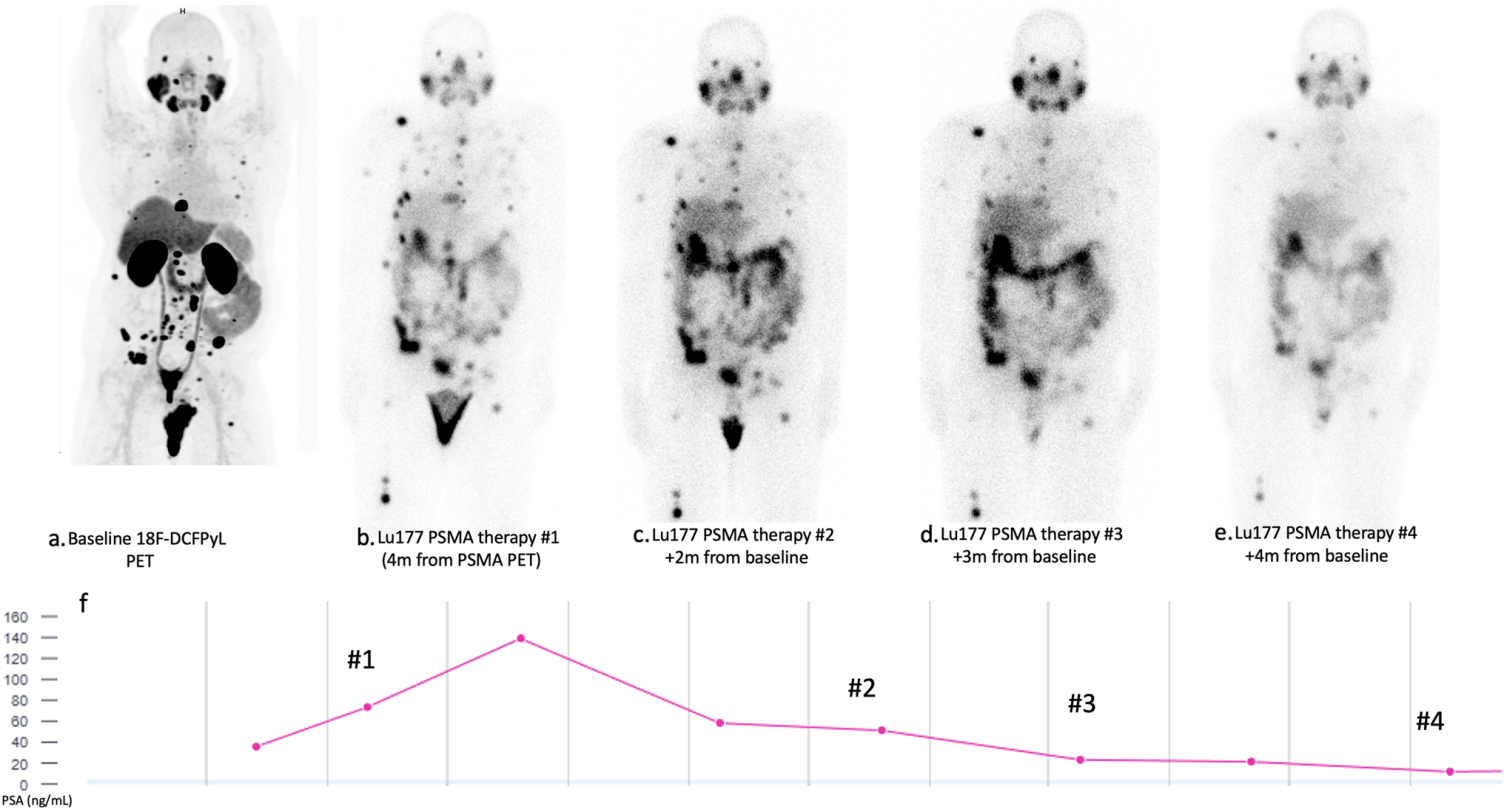
**a–e** 73-year-old with castration-resistant metastatic prostate cancer initially treated with RT and ADT in 2004, bone metastasis in 2019, treated with chemotherapy and RT to multiple lesions, started on 177Lu-PSMA treatment given recurrent disease and rise in PSA after baseline 18F-DCFPyL PET/CT (**a**). Subsequent post therapy whole-body uptake images (**b**–**e**) demonstrate gradual decrease in conspicuity of the osseous lesions, corresponding to interval treatment of the bone metastases. Overall decline in PSA levels was also noted during treatment (**f**). Maximum intensity projection 18F-DCFPyL PET **a** is scaled at 0–10 SUV

The life prolonging treatments for CRPC are diverse and include chemotherapy, hormonal therapy, immunotherapy and radioligand therapy (Fig 5). With recent approval from the food and drug administration on 177Lu-PSMA-617 (Pluvicto, 177Lu-vipivotide tetraxetan; Novartis [Basel, Switzerland]/Advanced Accelerator Applications USA, Inc. [Millburn, NJ]) based on the findings of clinical trials showing effectiveness of radiopharmaceutical therapy (RPT) [58, 59], PSMA imaging workgroup has recently updated the AUC to recommend PSMA imaging for evaluation of eligibility for patients being considered for PSMA-targeted RPT [60]. A recently published procedure guideline by EANM/SNMMI outlines the expert recommendations for patient selection, treatment protocol and management of side effects [61].

## Intraprostatic localization and pre biopsy evaluation

Tumor localization within the prostate can be helpful in biopsy targeting, targeted therapy and potentially eliminating the need for biopsy in patients with clinically insignificant prostate cancer. For the latter aim, PRIMARY trial [62] has been conducted which assesses the potential added value of PSMA PET to mpMRI in intraprostatic localization and possibly elimination of biopsies. A total of 296 patients with suspected prostate cancer without prior MRI or tissue sampling were included, then underwent MRI, biopsy and PSMA PET imaging. Combination of PSMA+MRI improved the negative predictive value to 91% versus 72% for MRI, improved sensitivity to 97% versus 83% while specificity decreased to 40% versus 53% [62]. The authors concluded 19% of patients could have potentially avoided biopsy by only risking 3.1% of patients with clinically significant prostate cancer. However, the MRI read at this study was not central and was comparable to similar studies with multiple central readers [63]. Therefore, further validation with larger sample size and potential incorporation of multivariate risk calculators would be warranted [64].

## PSMA in cancers other than prostate

**Fig. 6:**
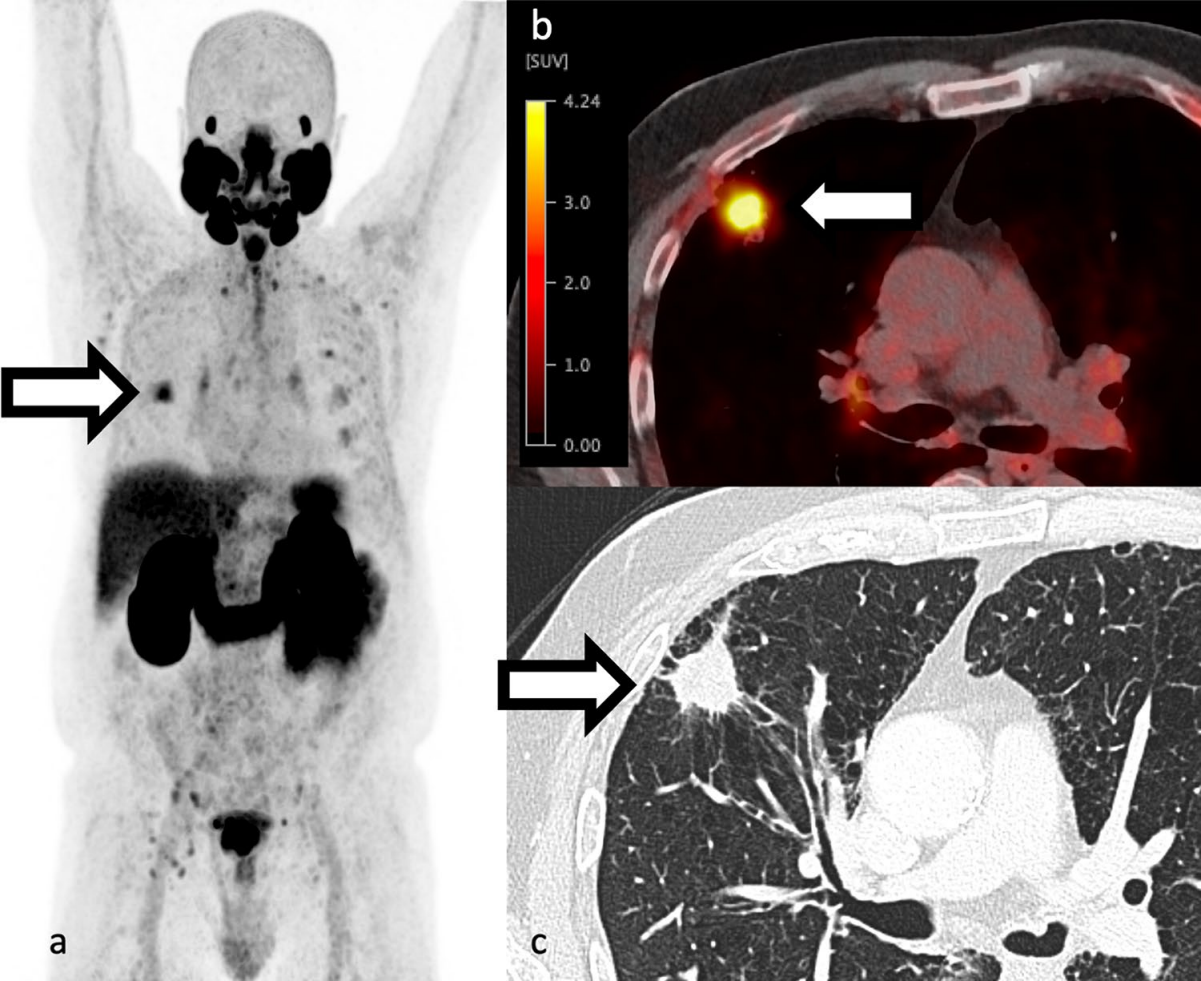
**a-c** 74-year-old man with a PSA 8.1, Gleason 4+3 prostate cancer. Staging Ga68-PSMA-11 PET findings consistent with T3bN1bMx disease and spiculated 2.6 cm right upper lobe nodule with low radiotracer uptake (SUVmax 5.8) on a background of emphysema (white arrow in panels a–c). Subsequent FNA was consistent with SCC of lung. There is increased uptake within the prostate with known cancer and metastatic pelvic lymph nodes. Several foci of low-level radiotracer uptake without CT correlate in the posterior left sixth rib without a CT or subsequent FDG correlate, favored to be benign. Maximum intensity projection **a** SUV scale 0–5

Despite the name, PSMA is not specifically expressed on prostate and a wide range of other cancers have PSMA uptake including renal cell carcinoma, transitional cell carcinoma, primary brain tumors, thyroid carcinoma, breast cancer, hepatocellular carcinoma, and lung (Fig. 6) [65]. Perry et al. retrospectively reviewed 1445 prostate cancer patients with atypical findings on 18F-DCFPyL PSMA PET and found non-prostate cancer tumors only in 1.2% of them with nearly all non-prostate cancer tumors showing no or low PSMA uptake, except renal cell carcinoma [66].

## Limitations of PSMA

Although PSMA-targeted imaging has been very effective in detection of PSMA expressing tumors, prostate cancers with neuroendocrine differentiation do not express PSMA due to FOLH1 gene suppression and thus, making this imaging technique ineffective [9]. Alternative molecular imaging targets including Fibroblast Activating Protein (FAP), Somatostatin receptor type 2, gastrin-releasing peptide receptor and FDG have been proposed as alternative diagnostic tests in these aggressive tumors [9, 67–69]. However, further studies are needed to show which, if any, of these alternatives are effective for imaging this subtype.

Androgen blockade is an integral part of the several treatment options for prostate cancer due to its significant role in improving survival rate and treatment effectiveness [70]. PSMA expression on prostate cancer cells is heavily modulated by treatments that alter androgen receptor [71, 72]. This response heterogeneity has implications for upcoming imaging and therapeutic interventions and differs depending on the tumor phenotype (hormone sensitive vs. CRPC) and the timing after treatment [71, 72].

**Fig. 7:**
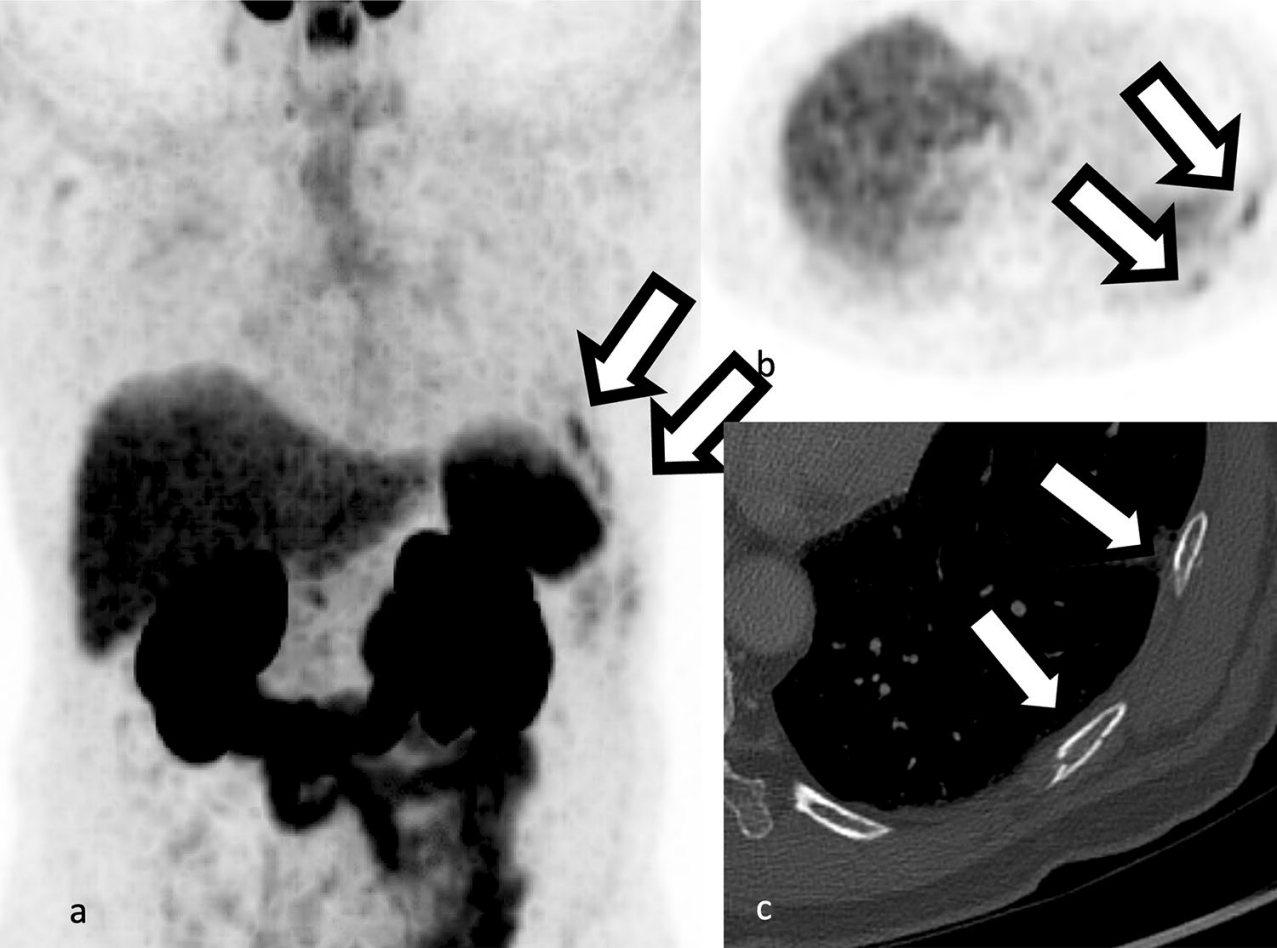
**a, b** 68Ga-PSMA-11 PET uptake due to fracture, better depicted on corresponding CT (**c**). Both a and b are scaled to 0–5 SUV.

Determination of malignancy or benignity of solitary bone lesions can be challenging in PSMA PET as this could change patient management and false positive interpretations would cause unnecessary harm to the patient [73]. Solitary rib lesions are common and mostly due to benign causes such as fracture or benign etiologies such as fibrous dysplasia (Fig 7). In a retrospective study of patients with prostate cancer, 62 patients with solitary Ga68-PSMA PET rib lesions were selected and their malignant potential was determined based on imaging follow up, PSA level and biopsy [73]. With these criteria, only one patient had a false negative finding of a rib lesion which resulted in worsening of metastatic disease and majority of lesions were classified as benign without need for follow up [73]. The findings of a recent study involving 48 high-risk primary or recurrent prostate cancer patients suggest that indeterminate 18F-DCFPyL PSMA PET bone lesions with a standardized uptake value maximum (SUVmax) exceeding 5 and coexisting bone metastasis in other areas are likely to be malignant, whereas lesions with SUVmax below 5 and no concurrent suspicious findings are more likely to be benign [74].

## Future directives

### PSMA PET imaging in metastatic-directed therapy of oligometastatic prostate cancer

**Fig. 8:**
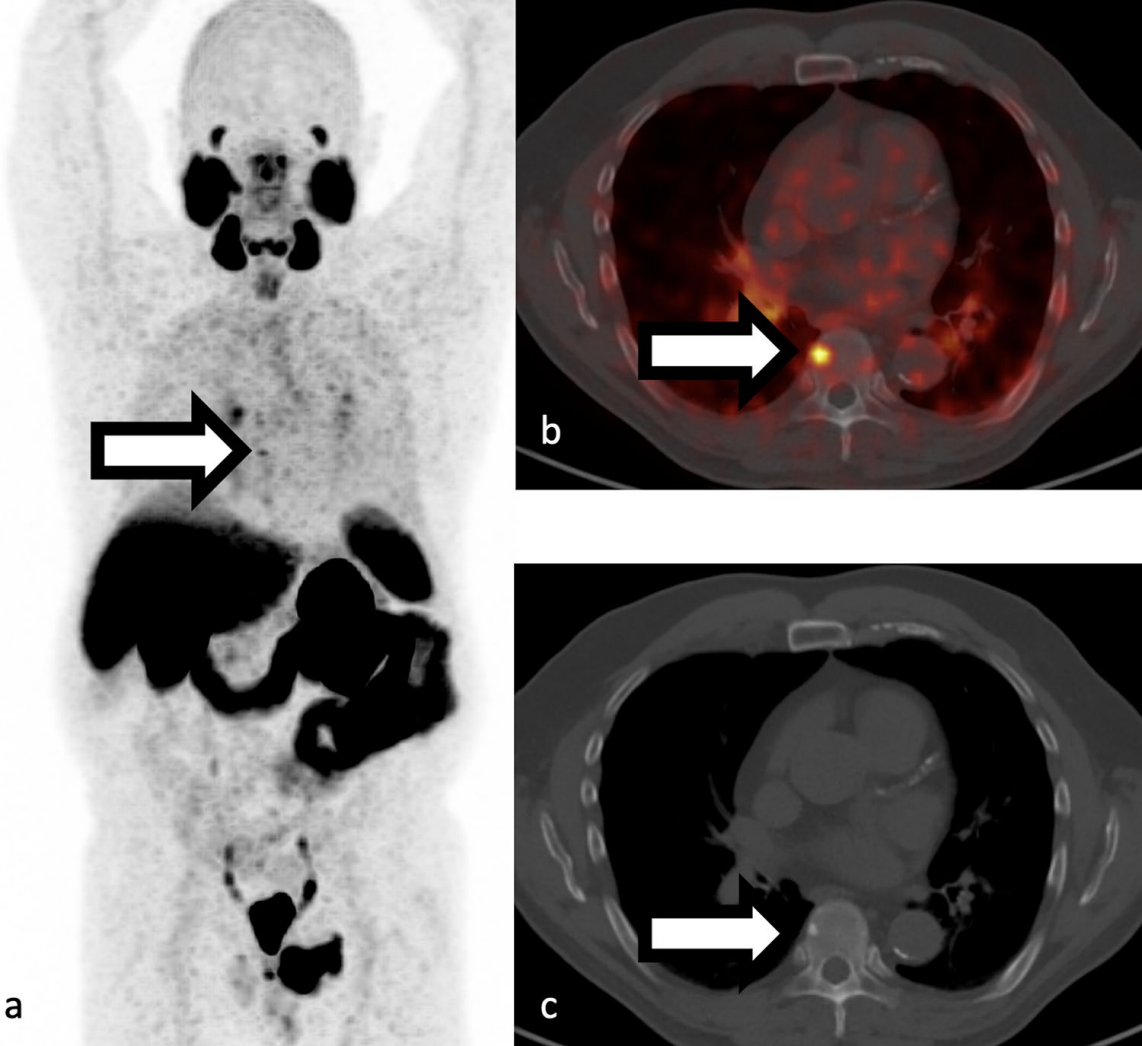
**a–c** Example of oligometastatic osseous disease in a 68-year-old man with history prostatectomy now presenting with biochemical recurrence and PSA of 1.3 ng/mL. 68 Ga-PSMA-11 PET/CT showed a single focus of PSMA uptake (white arrow in panels a and b, SUVmax 4) corresponding to a small sclerotic focus in the T8 vertebral body (white arrow in panel c). Activity below bladder corresponds to urinary contamination. Maximum intensity projection **a** SUV scaled at 0–5

The utilization of sensitive prostate-specific antigen (PSA) detection methods and improved PET radiotracers has resulted in an increased identification of “oligometastatic disease,” thereby raising interest in metastasis-directed therapy (MDT) [75, 76] (Fig 8).  Emerging evidence indicates that localized MDT may defer disease progression, prolong the time before systemic therapies become necessary, and mitigate-associated toxicities [75]. Multiple studies have studied the role of PSMA PET in treatment guidance of the oligometastatic prostate cancer with findings favoring improved progression-free survival [77]. A challenge in evaluation of the effectiveness of new more sensitive PSMA-directed oligometastatic disease treatment is the stage migration caused by improved sensitivity of these new techniques compared to conventional imaging which will limits use of historical data from previously performed trials [76].

### Assessment of response to treatment of CRPC using 177Lu-PSMA

In addition to its predictive role in determining the degree of response to 177Lu-PSMA therapy and aiding in patient selection, PSMA PET may also be utilized for evaluating treatment response [61]. The LuPIN trial investigated the response to 177Lu-PSMA-617 therapy and radiation sensitizer NOX66 in 37 patients with castration-resistant prostate cancer (CRPC). This prospective study employed 68Ga-PSMA and 18F-FDG PET scans both before and after treatment [78]. The study identified quantitative PSMA total tumor volume and PSA progression as the only two independently prognostic variables for overall survival among other variables [78]. Further studies with larger patient populations would be beneficial in further evaluating the role of post treatment PSMA PET as an imaging biomarker.

### Reporting frameworks for PSMA PET

PROMISE is a molecular imaging TNM staging system which is proposed to facilitate reporting and study design of prostate cancer molecular imaging [79, 80]. Under this system, local disease is categorized into T stages, ranging from miT0 (no local tumor) to miT4 (tumor invades structures other than seminal vesicles) [79]. Regional nodal disease is assigned N stages, ranging from miN0 (no regional lymph nodes) to miN2 (multiple pelvic nodal regions affected) [79]. Metastatic disease is classified with an M stage, which spans from miM0 (no distant metastases) to miM1 (distant metastases), further categorized as diss (disseminated), dmi (diffuse marrow involvement), oligo (oligiometastatic), and uni (unifocal) [79]. PSMA uptake levels are assessed using a visual scoring system based on the mean uptake in the blood pool, liver, and parotid gland, and the results are reported as 0, 1, 2, or 3, indicating no, low, intermediate, or high PSMA expression, respectively [79]. The recently introduced PROMISE V2 [80] comes with two hierarchical levels of assessment including an updated TNM staging schema and reporting of PSMA expression score which is now integrated with PRIMARY score [80]. PRIMARY score is a five-point scoring system, validated for detection of clinically significant prostate cancer in biopsy naïve patients [62]. PROMISE V2 has new recommendations for reporting sequential studies, lesion distribution and tumor volume assessment. These new changes facilitate use of specific PSMA response criteria such as PSMA PET Progression criteria (PPP) which focuses on a single lesion or Response Evaluation Criteria in PSMA-PET/CT (RECIP) which mostly focuses on total tumor volume in patients with extensive disease [62].

Prostate-specific Membrane Antigen-Reporting and Data System (PSMA-RADS) is a proposed reporting and data system with ultimate goal of establishing a uniform and structured reporting system for PSMA-targeted PET studies [81]. This framework assigns a PSMA-RADS value of 1–5 to both individual lesions and the overall scan. A value of 1 indicates a normal scan or a known benign lesion based on pathological examination or pathognomonic imaging. A value of 2 suggests a likely benign lesion, although biopsy results may be unavailable, or imaging findings may not be pathognomonic. Category 3 is multifaceted and includes lesions that are indeterminate for prostate cancer or suspicious for a non-prostate malignancy. A value of 4 indicates a likely prostate cancer, while 5 signifies almost certain prostate cancer with a classic imaging appearance [81]. The aim of this framework is to reflect the level of confidence of the clinician in presence of prostate cancer and clarify the need for further workup. Different categories of PSMA-RADS specifically category 3 which is reflects more equivocal findings, has been validated in recent studies [82–84].

## Concluding remarks

PSMA PET is considered a valuable tool for various aspects of patient care in the clinical management of prostate cancer and has shown promising results in initial staging of prostate cancer, localization of recurrent or persistent disease, and staging before PSMA-directed radioligand therapy. It also has potential applications in guiding prostate biopsy, guiding metastatic-directed therapy, and monitoring systemic and radioligand treatment response. While the impact on patient outcomes and management is still being assessed, PSMA PET has been included in clinical guidelines and consensus documents, highlighting its superior accuracy and additional value in prostate cancer staging. However, further research and evaluation are needed to fully establish its role in treatment monitoring and patient outcomes.
